# Exploring Medication Safety in Transitions From Prison to Community: A Qualitative Study

**DOI:** 10.1111/hex.70684

**Published:** 2026-05-03

**Authors:** Claire Planner, Richard N. Keers, Denham Phipps, Ozlem Eylem van Bergeijk, Natasha Tyler, Jennifer Shaw, Darren Ashcroft, Maria Panagioti

**Affiliations:** ^1^ NIHR School for Primary Care Research, Division of Population Health, Health Services Research and Primary Care, School of Health Sciences, Faculty of Biology, Medicine and Health The University of Manchester Manchester UK; ^2^ NIHR Greater Manchester Patient Safety Research Collaboration University of Manchester Manchester UK; ^3^ Division of Pharmacy and Optometry, School of Health Sciences, Faculty of Biology, Medicine and Health The University of Manchester Manchester UK; ^4^ Optimising Outcomes with Medicines (OptiMed) Research Unit Pennine Care NHS Foundation Trust Manchester UK; ^5^ Division of Psychology and Mental Health, School of Health Sciences, Faculty of Biology, Medicine and Health The University of Manchester Manchester UK

**Keywords:** care transitions, continuity of care, medication safety, prison release, professional perspectives

## Abstract

**Background:**

The prison population in England and Wales exceeds 88,000, with a high turnover – 47% of sentenced admissions in 2023 served less than 12 months. Transitions from prison to the community are recognised as high‐risk periods for medication‐related harm, driven by complex health needs, short custodial stays, and fragmented healthcare systems. While national and international guidance exists to support safe medication management, implementation during prison‐community transitions remains inconsistent, and evidence on both the drivers of unsafe medication practices and potential solutions is limited.

**Aim:**

This study explored the human, organisational, and environmental factors influencing medication safety during transitions from prison to the community, as well as potential solutions for improvement, from the perspective of staff involved in these transitions.

**Methods:**

Qualitative semi‐structured interviews were conducted with 12 staff members working in roles relevant to transitions from prison to the community, including general practitioners, pharmacists, and prison officers. Participants were recruited through professional networks and snowball sampling. Data were thematically analysed using the Systems Engineering Initiative for Patient Safety (SEIPS) framework.

**Results:**

Five main factors impacting medication safety during transitions were identified: release practices, care coordination and communication issues, staffing shortages, IT system limitations, and patient‐related factors. Key findings highlighted risks associated with immediate releases, discontinuity in medication regimens, insufficient staffing for discharge planning, and poor information transfer between prison and community healthcare providers. These challenges were further compounded by patient‐level issues such as low health literacy, substance use, and housing instability. Staff proposed several improvements to enhance medication safety during prison‐to‐community transitions, including electronic prescribing for timely access to medication, improved information transfer, dedicated discharge teams to ensure medication follow‐up, early discharge planning to address medication needs, and multi‐disciplinary meetings to coordinate complex care.

**Conclusion:**

Medication safety during transitions from prison to community healthcare requires coordinated efforts to address organisational challenges, including short‐notice releases and inadequate information transfer, as well as human factors such as communication barriers and staffing constraints. Improvements that clarify roles, enhance processes and technology, and foster cross‐system collaboration are essential to ensuring continuity of care and medication safety.

**Patient or Public Contribution:**

Two people, one with lived experience of care transitions and one carer, contributed to study design, recruitment strategies, participant materials, and the analysis plan through quarterly input. Findings were shared with a wider group of lived experience representatives, carers, professionals, and policy makers, who informed interpretation and dissemination. While PPI members did not directly participate in coding or analysing the data, their input ensured that the study design and interpretation were informed by real‐world perspectives.

## 1 Introduction

With over 88,000 inmates, the prison population in England and Wales faces complex healthcare challenges, exacerbated by high turnover – 47% of sentenced admissions in 2023 were for under 12 months [1, 2, 3, 4]. Up to two‐thirds of people leaving prison have physical or mental health conditions, substance misuse disorders, or multimorbidity, with mental illness up to ten times more prevalent than in the general population [5, 6, 7]. Suicide rates have risen by 26% in the year to June 2023, and nearly half of all prisoners are in treatment for drug or alcohol dependency, often involving opiates [8, 9]. Many also face low health literacy and socio‐economic disadvantage, making safe and effective medication management essential [10, 11].

Medication‐associated harm is a major source of avoidable harm globally, and evidence of its impact in prisons is growing [12, 13]. In response, the World Health Organisation (WHO) launched Medication Without Harm in 2017 to halve such harm by 2024 [14]. Despite recognition of these risks, there remains limited evidence on the causes of unsafe medication practices—particularly during transitions from custody to community [15]. These high‐risk periods are marked by fragmented care, poor communication, and post‐release instability, such as homelessness and financial insecurity [16, 17, 18].

NHS England in the UK has advised close coordination between general practices and community pharmacies to ensure smooth healthcare transitions and safe integration for people released from prison [19]. Yet, people in prison are often deregistered from general practice and may not re‐register on release, disrupting continuity of care [20, 21]. The WHO identifies poor information flow between services as a key driver of medication errors, and the Royal College of General Practitioners in the UK recommends timely communication of discharge information and provision of a 7‐day supply of medication, including controlled drugs [22]. However, implementation remains inconsistent, and data on pre‐release medication reviews are incomplete [23, 24].

A recent large‐scale quantitative analysis of prison patient safety reports in England found that one in ten incidents occurred during care transitions—mostly medication‐related—due to poor continuity and failures to follow protocols [25]. Complementing these findings, a retrospective case note review across 18 English prisons identified 247 cases of avoidable healthcare‐associated harm, with an incidence rate substantially higher than that observed in community settings, and many cases involving delays in appropriate management and moderate to severe harm [26]. Moreover, Building on this, the current qualitative study explores prison healthcare staff's perspectives on the human, organisational, and environmental factors that influence medication safety during transitions from prison to community, and aims to identify practical strategies to reduce associated risks.

## 2 Methods

The study was conducted following the Consolidated Criteria for Reporting Qualitative Research (COREQ) [27], and a completed COREQ checklist is included in the supplement (Appendix 1).

### 2.1 Research Design and Setting

This qualitative study employed semi‐structured interviews to explore factors influencing medication management during the transition from prison to the community. In this study, care transitions refer specifically to the transfer of healthcare from prison‐based services to community healthcare at the point of release, rather than transfers between prison and hospital care during incarceration. Prison healthcare staff participants were asked to reflect on the experiences and needs of people leaving prison and returning to the community. The aim was to capture participants' experiences in depth, highlighting both system‐level and individual‐level influences on medication safety. The study was informed by a pragmatic qualitative epistemology, recognising that participants' accounts reflect situated professional experiences shaped by organisational and system contexts, and that these perspectives can inform practical improvements in care.

### 2.2 Patient and Public Involvement (PPI)

Two PPI contributors, one with lived experience of care transitions and one carer, were involved from the outset. They provided input on study design, recruitment strategies, and participant materials, ensuring that the study addressed relevant real‐world issues. Their guidance informed the structure of the interview guide and helped refine prompts to reflect practical concerns in medication management. PPI Reporting followed the GRIPP2 checklist to ensure transparency and quality [28].

### 2.3 Recruitment and Data Collection

Participants were recruited using a combination of staff contacts, professional networks, and snowball sampling to broaden reach across relevant professional roles. PPI input contributed to refining recruitment materials and participant information, helping ensure clarity and accessibility. While this approach facilitated access to participants with direct experience of the prison–community transition, it may have introduced recruitment bias by favouring individuals engaged in professional networks or interested in medication safety.

Data collection took place between July 2022 and January 2023. Potential participants were provided with a detailed information leaflet outlining the study's purpose, procedures, confidentiality arrangements, and expectations, alongside a consent form. All participants provided recorded verbal consent prior to taking part in online interviews. Ethical approval was granted by the University of Manchester Research Ethics Committee (UREC).

Interviews were guided by a topic framework structured around three core areas (see Appendix 2): medication safety issues and their perceived causes; ‘work as imagined’ (how tasks are intended to be performed according to policy or guidance) versus ‘work as done’ (how tasks are carried out in practice); and potential points of failure. Prompts were informed by the Systems Engineering Initiative for Patient Safety (SEIPS) framework [29], enabling exploration of interactions between system elements, human behaviour, tools, tasks, and organisational context. The SEIPS framework therefore provided a theoretical lens guiding both data collection and subsequent analysis. Interviews were conducted online via Microsoft Teams by CP, lasting between 34 min and 1.5 h (mean: 56 min), audio‐recorded, and transcribed verbatim by a University of Manchester–approved service.

Participant confidentiality and anonymity were prioritised throughout the study. Transcripts were anonymised at the point of checking, with all identifying information removed. Participants were assigned unique identifiers, and data were stored securely on password‐protected university servers accessible only to the research team.

CP reflected on their positionality as a researcher with experience in patient safety and healthcare research, which may have influenced the framing of questions and interpretation of participants' accounts. Reflexive notes were maintained during and after interviews, informing analytical decisions and supporting consideration of alternative interpretations.

### 2.4 Data Analysis

Data were analysed using thematic analysis [30], employing a primarily deductive approach guided by the SEIPS framework [29], alongside inductive coding to capture unanticipated issues emerging from the data. PPI input was used to review preliminary findings, supporting interpretation and ensuring that emerging themes were meaningful and aligned with real‐world experiences, although PPI members did not directly code or analyse transcripts.

All transcripts were checked for accuracy, anonymised, and read repeatedly to ensure familiarity. Line‐by‐line coding was conducted by the first author (CP) using NVivo. An initial coding framework was developed and iteratively refined throughout the analysis. Regular research team meetings were held to discuss emerging findings and interpretations, with differences explored through discussion and consensus‐building.

Codes were grouped into themes and sub‐themes, which were reviewed and refined to ensure they accurately reflected the dataset and addressed the study aims. Key illustrative quotations were extracted and managed in an Excel workbook to support transparency.

The study involved a relatively small sample size, reflecting challenges in recruiting a hard‐to‐reach professional group within a limited timeframe. However, ongoing analysis indicated that no new themes were emerging in later interviews. Data saturation was assessed through iterative comparison of codes and themes across interviews.

## 3 Results

Twelve participants were recruited from various roles across community healthcare and prison settings, including GPs, pharmacists, and prison officers (see participant details in Appendix 3).

**Figure 1:**
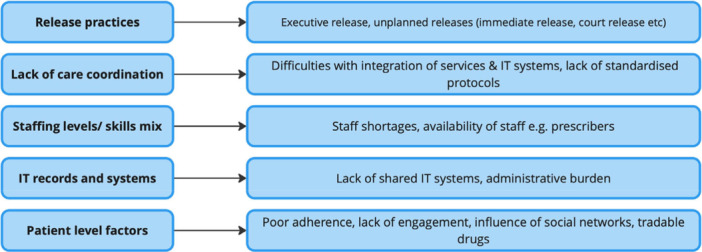
Contributory factors to medicine management issues. *Note:* Factors contributing to medicines management issues.

Five main factors were identified as contributing to medication safety incidents or posing potential threats to medicines continuity during transitions from prison to the community (see Figure 1).

1. **Release practices**

Participants highlighted major challenges with immediate and court releases, especially on evenings, Fridays, weekends, or bank holidays when community healthcare services were limited or closed. If prescribers based in prison were unavailable to arrange discharge prescriptions, this could lead to individuals leaving prison without medication.

…. they've [the prisoner] got the order for executive release [(i.e., release authorised by a senior prison or government authority rather than through routine parole], they will demand that they're escorted out of the prison immediately. So, I think that's normally a risk. And, yeah, there are cases where patients do go without medication.

(P1)

One of the problems we have is the immediate releases. So this can be, e.g., somebody six o'clock in the evening, they've gone to court, back to us in prison and then they get told they're going…. so they will go out with no prescriptions. It's literally “you're going now”. So that can be a problem.

(P12)

So we have had patients where they've ended up missing a day's worth of meds [methadone] because they've [prison staff] just not been able to get the prescription to the team in time or physical medications out in time.

(P11)

Participants discussed workarounds, for example, for people on methadone, arranging emergency appointments with community drug teams or delivering prescriptions directly to community pharmacies.

2. **Lack of care coordination at the prison community interface**

All participants reported significant gaps in care coordination between prison and primary healthcare services, which sometimes leads to disruptions to medication regimens. Prison staff described the prison setting as unique because it's a controlled environment where medications can be initiated, tapered or stopped, health assessments conducted, and structured programmes for addiction and education delivered. Participants working in prisons expressed frustration that carefully managed treatments could be interrupted after release due to systemic failures across multiple elements of the healthcare system — including people, tasks, tools and technologies, and organisational structures — as conceptualised within the SEIPS framework.

We [prison healthcare staff] may have commenced medication treatments and then that patient goes out into the community and goes see their GP and their GP says “no, I'm not continuing with this”.

(P3)

…it's really hard for us because sometimes that patient will then bounce back into us and we're like, we've spent 4 months trying to get you off this, you've gone back, asked the GP and they've just re‐prescribed it.

(P11)

Additionally, coordination with secondary care or other specialist services (e.g., HIV treatment, alcohol dependency etc) could be lost upon release, further compromising continuity.

Human factors such as limited communication between prison staff, community providers and the patient, and organisational issues including fragmented patient registration systems, lack of integration of IT systems created barriers to coordinated care.

3. **Staffing issues**

Some participants highlighted staffing shortages as a barrier to effective discharge planning and medicines management. Limited personnel reduced the time available to coordinate safe transitions, impacting the quality and continuity of care. Organisational ambiguity about roles further complicated this issue—there was no clear agreement on who should lead medicines management at discharge. Suggestions varied, including nurses, pharmacy technicians, or dedicated discharge teams, reflecting unclear task allocation and coordination within the system.

There's not a dedicated team just to deal with releases. And I think that's probably our downfall. That process can be quite rushed and usually it's dependent on people to…who are free to be able to do that.

(P4)

I would love to see that pharmacy role really expand, because nurses are very good at discharging. We're very good at that process, ticking this and okay, this is in place, but pharmacy to me is pharmacy, it's medications. Now, I would like to see that those pharmacy teams I think are the missing link. I think they don't see themselves as part of that discharging team.

(P2)

4. **Transfer of Information at discharge**

Participants across all professional groups highlighted IT and information transfer at discharge as a major challenge affecting medication safety. A lack of records systems integrations impacts information continuity for example, when prison staff to access post‐release healthcare records limits their capacity to monitor medication continuity.

They'll never have seen them before. There's been many times in community where I've been working when they have gone to see their GP, they've been prescribed medication that's contraindicated if they're on methadone, the GP wasn't even aware they were on methadone. So, yeah, it's difficult. So, I think that's a community issue that they're trying to sort out as well.

(P4)

Once they're released from our system, they're off. So they are deducted from our SystmOne module so we can't see [their records]. So unless, a GP surgery might ring us to clarify what's happened, or unfortunately the patient may come back into custody, we just don't know. So in that sense it's sad really, because there's not that continuity, the GPs can't see what's happening within our setting.

(P3)

Some medications will be stopped in prison, and it hasn't been explained to them [patient] as to why it's been stopped, and if we don't have that information either, it's a bit of a puzzle.

(P6)

We don't know where to send the discharge summary. So, there is a bit of…I think there's a critical period there, if they are on any critical meds, potentially they've left [prison] without them.

(P1)

Although electronic systems like ‘GP2GP’ support secure data transfer between prison and primary care, some participants reported issues with the volume and complexity of shared information, delays, and errors, sometimes necessitating time‐consuming data review.

So they're making sure that stuff links up. And that stuff when it's pulled through is appropriate. So for example, sometimes it will pull through really weird allergies. Or that their allergies don't quite match what's actually already in our portal. And sometimes it's just the way it's been read coded. So there's quite a lot of data cleansing that needs to happen from an admin side as well, which is very heavy on our teams.

(P3)

But that GP2GP, there's a delay sometimes and you get a basic letter or you get basic information. The rest will populate in a week or so, but that's not going to help you if you're going to try and sort your patient out.

(P8)

5. **Patient factors**

Participants identified various patient‐level factors influencing medication safety during transitions, including mental health challenges, treatment non‐adherence, limited engagement with staff, low health literacy regarding medication regimens, insecure housing or homelessness, and the role of social networks.

GP participants described disengagement upon release and the problems it can present in terms of maintenance treatments:

They don't present, and if they've had a few days where they haven't had a dose, that we, obviously more than 3 days in a row, we're having to start from scratch again.

(P9)

Often, they won't attend, which will be a bit of a problem, because when you do eventually see them, because they obviously need to be on this medication, so they will eventually make contact. You're just having to go from the start.

(P6)

Non‐adherence can be complex especially when involving substance dependence:

They will go and pick up the methadone script and not pick up anything else.

(P7)

Health beliefs are also a factor:

…it depends on how high up their own personal health beliefs, they hold their medication. And for some of them, they just want to be released. So if there's a delay in the sense that, the nurse needs to go through things with them at release or, sometimes they'll just go and they'll say, I'm not waiting.

(P3)

Along with non‐medical issues

housing, that's the most…and just getting settled again in an area, getting their bank account sorted, so you often deal with all those other things that come part and parcel, often they'll have chaotic lives, and put in hostels, maybe, as a temporary solution, which is not great, because a lot of service users are based in hostels. There's a lot of drug substance misuse in hostels, so if you're trying to recover, it's not the best place for you to be.

(P6)

These Patient factors sometimes interacted closely with organisational and environmental elements. For example, delays in medication availability at release often led to frustration and disengagement, highlighting the dynamic interplay between patient needs, staff actions, and system processes.

**Figure 2:**
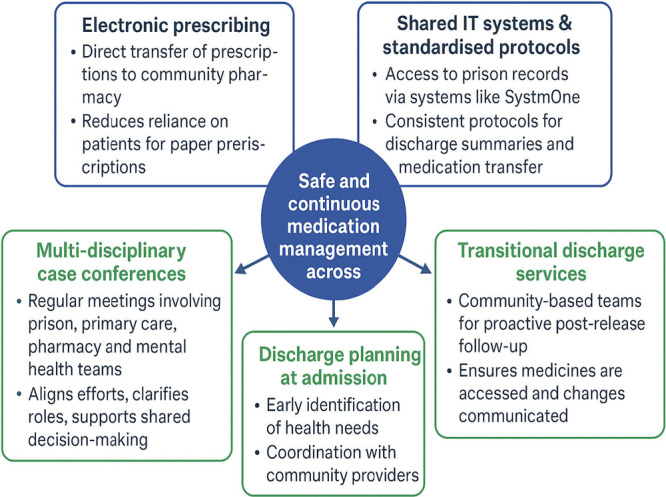
System‐level improvements to better support medicines management during transitions from prison to community.

Participants suggested several system‐level improvements to better support medicines management during transitions from prison to community (see Figure 2). These suggestions reflected how different work system elements—such as tools, tasks, people, and the organisational environment—could be reconfigured to enhance safety and continuity.

1. **Electronic prescribing to improve continuity**
  Some participants suggested that electronic prescribing, which would allow prescriptions to be transferred directly to a named community pharmacy would reduce reliance on patients managing paper prescriptions and allow prison staff to track whether medicines had been collected post‐release—enhancing information continuity across settings.
  It's the way the prison system's set up. We don't have access to electronic prescribing. If we had access to that, it would open up another window for us to be able to capture these patients, because you can electronically prescribe the FP10s to go directly to a pharmacy, and you can track whether that prescription has been dispensed as well and picked up by the patient.
  (P5)
  …the system, the prison system, this not just a company thing it's the Health and Justice as a whole, we don't have access to that electronic prescribing the way you would in community.
  (P8)
2. **Shared IT systems and standardised protocols**
  Shared access to healthcare systems such as SystmOne was viewed as a potential enabler of safer transitions, allowing community providers to view prison records and treatment decision
  It's really about making sure the information about what's happening during their stay, is getting out [to community health care providers], we're not quite there yet…GP2GP was meant to solve a lot of problems, and it hasn't really.
  (P3)
  So the ideal situation is there's full transparency [through shared systems] because that would actually alleviate some of the safety issues that we have when we are releasing people into the community.
  (P3)
  Participants also emphasised the need for consistent protocols for transferring discharge summaries and medication information, to reduce variability and to improve the quality of information shared across the prison/primary care interface.
  Sometimes those letters that come out, they're so basic. Like, literally, this guy has got released out of prison, he's now in community care, he's under the drug team, gets 50 ml of methadone, this is the rest of his medication, full stop……I barely get a paragraph.
  (P5)
3. **Establishing transitional discharge services**
  The absence of clear post‐release follow‐up was seen as a gap in the system. Participants advocated for community‐based teams who could proactively follow up with individuals after release, ensuring medicines had been accessed and changes communicated across services. Such roles could bridge prison and primary care systems, improving coordination across organisational boundaries.
  To be honest I think taking that process out and having its own dedicated clinic for discharges is probably what's needed. That would be I think a gold standard. Because then you've got a dedicated team to be able to then follow them up in the community just to ensure they have engaged with a practice or they are in good health.
  (P4)
  We were discussing a discharge service that sits outside of the prison service. it's more like a GP practice that its sole responsibility is to ensure everyone who's getting released has their medication reviewed and FP10s potentially prescribed. So, that that workload is taken off the staff on site [prison].
  (P5)
4. **Discharge planning at admission**
  Some participants felt discharge planning should begin at the point of prison entry. Early identification of health needs, coordination with community providers, and advance planning for medication adjustments were seen as ways to reduce last‐minute delays and ensure smoother transitions.
  I think definitely from a prison point that prisoner having a named nurse. People band that around, and that doesn't really happen a lot. Where actually we start to plan discharge on admission. That discharge can be 6 months or 12 months or it can be 2 weeks, but start to plan it on admission.
  (P2)
5. **Using multi‐disciplinary case conferences to improve communication**

Participants identified fragmented communication across services as a persistent barrier. Regular case conferences involving prison, primary care, pharmacy, and mental health teams were suggested as a way to align efforts, clarify roles, and enhance shared decision‐making—particularly for individuals with complex needs.

I think personally with that I would love to see, in a beautiful ideal world, that with Skype and Teams, hybrid that we would be involved in the GP, in the community, in that discussion [around transition planning]. Now, that to me would resolve a lot of those problems.

(P10)

## 4 Discussion

### 4.1 Summary of Key Findings

This study provides novel insights from prison healthcare staff perspectives on how medication safety during transitions from prison to community healthcare is influenced by interactions between human, organisational, and technological factors across the release journey. Professionals reported that early discharge planning—beginning at prison entry—is rarely implemented, despite its potential to support safer prescribing and timely access to medication. Staffing shortages and unclear responsibilities for medication management further limit the effectiveness of discharge processes.

At the point of release and immediately afterward, professionals highlighted persistent system‐level gaps in information transfer and IT integration, including inconsistent sharing of discharge summaries and limited interoperability between prison and primary care records. These gaps led to delays in medication continuity and placed additional burdens on both patients and providers. Participants proposed specific interventions—such as electronic prescribing, shared access to medical records, transitional follow‐up services, and multi‐disciplinary case conferences—which collectively reflect practical, implementable strategies to strengthen medication safety.

By focusing on professional experiences and observations, this study extends existing literature by identifying concrete, multi‐level opportunities for intervention and highlighting how patient‐level vulnerabilities interact with organisational weaknesses. Our findings emphasise that improving medication safety requires coordinated action across professional roles, processes, technologies, and organisational structures, rather than relying solely on individual patient adherence.

### 4.2 Comparison With Existing Literature

This study adds to a limited evidence base by examining medication safety specifically during the transition from prison to community healthcare, from the perspective of service providers. Our findings align with previous research on medication and patient safety within prison settings, which highlights the complexity of ensuring safe medication management due to factors such as governance structures, workforce challenges, IT limitations, polypharmacy, and the tradability of medications [13, 15, 25]. Variability in prison practices and transitions of care have also been identified as particularly high‐risk periods.

Our findings also resonate with a recent study exploring the implementation of prescribing safety indicators via a multi‐disciplinary intervention across 30 English prisons [31]. That study similarly emphasised the importance of collaborative working, integration with existing safety practices, and clear roles to support successful implementation. Despite challenges such as limited staff capacity and competing priorities, participants acknowledged the benefits of structured, system‐level approaches to improve prescribing safety. These findings reinforce our conclusion that improving medication safety during high‐risk transitions requires coordinated reforms at both systemic and local levels, tailored to the operational realities of prison healthcare.

Medication safety interventions could be embedded within broader release planning efforts, as demonstrated in hospital settings. A recent scoping review identified promising interventions—such as peer support (‘prison buddies’), pre‐release training, and personalised pathway documents—that address wider social care needs after release [21]. This aligns with evidence showing that continuity of care between prison and community settings is critical for improving health and social outcomes, including reductions in re‐offending among people released from prison [32, 33]. Incorporating patient‐centred medication support within these frameworks could further strengthen continuity of care and reduce risks post‐release.

Across studies, proposed solutions consistently emphasise the need for multi‐disciplinary collaboration, greater standardisation of medication‐related processes, and enhanced staff training and support. Taken together, the evidence highlights the importance of developing interventions that are context‐sensitive, sustainable, and system‐wide. Behaviour change models such as COM‐B may provide a useful foundation for designing interventions that address staff capability, opportunity, and motivation to deliver safe and effective medication practices in the complex environment bridging prison and community healthcare [15].

## 5 Limitations

This study provides novel insights into medication safety during transitions from prison to community healthcare, but several limitations should be acknowledged. The sample size was relatively small and recruitment relied on snowball sampling, which may have introduced selection bias and limited generalisability beyond the participants' settings and experiences. Although ongoing analysis suggested that no new themes emerged in later interviews, it is possible that additional perspectives were not captured. While the sample included participants from a range of professional roles, it may overrepresent individuals with a particular interest in medication management. We collected information only professional roles, which—while appropriate for exploring system‐level perspectives—limits understanding of how factors such as experience, gender, or type of prison setting may shape views. The analysis was led by a single researcher, with emerging findings discussed and refined through regular research team meetings rather than independent double‐coding of transcripts. While this approach supported reflexivity and analytical coherence, alternative interpretations of the data may exist. In addition, the exclusive focus on healthcare professionals' perspectives limits understanding of the transition experience from the viewpoint of people leaving prison. While PPI contributors informed the study design, recruitment strategies, materials, and interpretation of findings, they did not directly participate in data collection or analysis, and direct input from people with lived experience was not captured. Including the voices of those directly affected would provide a more holistic understanding of the practical challenges, priorities, and unmet needs during transitions and could help identify additional opportunities for improving care. Future research should actively engage people with lived experience to complement professional perspectives and ensure proposed solutions are responsive to the needs of service users. Finally, the study relied on self‐reported accounts rather than direct observation of discharge planning or transition processes; ethnographic approaches could offer richer, real‐time insights into the practical complexities of medication management [34, 35]. Participants described unclear responsibility for discharge medication and fragmented data systems, but practices and systems vary nationally. Future research should explore this variability and identify examples of good practice across different settings.

### 5.1 Implications for Policy and Practice

To improve medication safety during transitions from prison to community healthcare, discharge planning should begin earlier—ideally at prison entry—and responsibilities for medication management need to be clearly defined. Integrated IT systems and consistent information sharing between prison and community healthcare are essential to reduce delays in post‐release prescribing. Supporting individuals through transitional services, such as medication reviews or follow‐up contacts, could ease the burden on patients and providers. Embedding medication support within wider release planning, alongside addressing social needs, will help maintain continuity of care and prevent avoidable medication‐related harm during this critical period. However, implementing these solutions may be challenging due to limited staff, resources, IT compatibility issues, and the need for coordination between prison and community services. Strong organisational commitment is needed to make these changes effective and maintain continuity of care.

## 6 Conclusion

This study found that safe medication practices during the transition from prison to community healthcare require coordinated action across the whole system. Organisational challenges—such as short‐notice releases, unclear staff roles, gaps in information sharing, and poor coordination between prison and community healthcare—are closely linked with human factors like communication problems, heavy workloads, and differences in staff training and roles. Addressing these, along with practical and social barriers such as low health literacy and unstable housing, is essential to improving medication safety during this high‐risk time. Effective solutions should create processes and use technologies that support staff, clarify responsibilities, and encourage teamwork across organisations to ensure continuity of care and medication safety.

## Author Contributions

**Claire Planner:** conceptualisation, writing – original draft, formal analysis, methodology, writing – review and editing, project administration. **Richard N Keers:** conceptualisation, methodology, writing – review and editing, funding acquisition. **Denham Phipps:** conceptualisation, methodology, supervision, writing – review and editing, funding acquisition. **Ozlem Eylem van Bergeijk:** writing – review and editing, validation, methodology. **Natasha Tyler:** conceptualisation, methodology, writing – review and editing, writing – original draft. **Jennifer Shaw:** conceptualisation, funding acquisition, supervision, resources, writing – review and editing. **Darren Ashcroft:** conceptualisation, funding acquisition, writing – review and editing, supervision. **Maria Panagioti:** conceptualisation, investigation, funding acquisition, writing – original draft, writing – review and editing, methodology, supervision.

## Conflicts of Interest

The authors declare no conflicts of interest.

## Supporting information

Supporting File 1:

Supporting File 2:

Supporting File 3:

## Data Availability

The data that support the findings of this study are available on request from the corresponding author. The data are not publicly available due to privacy or ethical restrictions. This is a qualitative study confined to relatively small groups of health care professionals in specific roles. Making the full transcripts publicly available could therefore potentially lead to the identification of participants. Our ethics approval was granted based on the anonymity of the individuals consenting to participate and specifically referred to only anonymised quotations being used in reports. As such the participants did not consent to full their transcript being made publicly available. Therefore, full transcripts cannot be made publicly available, however detailed codes, themes and full extracts from transcripts can be made available on request.
