# Supplementary material for: Exploring Medication Safety in Transitions From Prison to Community: A Qualitative Study
Source: Health Expect. 2026 May 3;29(3):e70684. doi: 10.1111/hex.70684 (PMC13136601; doi:10.1111/hex.70684)
Supplement: Supplementary file 1 — Supporting File 1: [file HEX-29-e70684-s003.docx]

**COREQ 32-Item Checklist**

| **Domain** | **Item** | **Reported in Manuscript (Page/Section)** |
| --- | --- | --- |
| **Research team and reflexivity** |  |  |
| 1 | Interviewer/facilitator | Methods – Research design and setting: “CP (first author) conducted all interviews” |
| 2 | Credentials | Title page / Methods |
| 3 | Occupation | Title page / Methods: team includes clinicians, pharmacists, academics in patient safety and prison healthcare |
| 4 | Gender | Not collected (Methods – Participant characteristics) |
| 5 | Experience and training | Methods – “First author experienced in patient safety research; reflexive notes maintained” |
| 6 | Relationship established | Methods – “No prior relationship; rapport built at start of interviews” |
| 7 | Participant knowledge of interviewer | Methods – participants informed of study aims and interviewer’s professional role |
| 8 | Interviewer characteristics | Methods – positionality and reflexivity discussed |
| **Study design** |  |  |
| 9 | Methodological orientation | Methods – “Pragmatic qualitative epistemology; SEIPS framework guided interview prompts and analysis” |
| 10 | Sampling | Methods – “Purposive and snowball sampling to reach professional roles” |
| 11 | Method of approach | Methods – “Recruitment via staff contacts, professional networks, snowball sampling” |
| 12 | Sample size | Results – “12 participants from prison and community healthcare roles” |
| 13 | Non-participation | Not explicitly reported |
| 14 | Setting of data collection | Methods – “Online interviews conducted remotely” |
| 15 | Presence of non-participants | Methods – “Only participant and interviewer present” |
| 16 | Description of sample | Methods / Results / Appendix 2 – roles and professional background described |
| 17 | Interview guide | Methods – topic guide informed by SEIPS framework and PPI input |
| 18 | Repeat interviews | Methods – single interview per participant |
| 19 | Audio/visual recording | Methods – “All interviews were audio-recorded” |
| 20 | Field notes | Methods – “Reflexive notes maintained during and after interviews” |
| 21 | Duration | Methods – 34 mintues to 1.5 hours |
| 22 | Data saturation | Methods – “Ongoing analysis indicated no new themes emerging” |
| 23 | Transcripts returned | Methods – transcripts anonymised; participants not returned transcripts |
| **Analysis and findings** |  |  |
| 24 | Number of coders | Methods – “Primary coding by CP; regular team meetings to discuss themes” |
| 25 | Description of coding tree | Methods – “Coding framework iteratively developed; themes and sub-themes refined” |
| 26 | Derivation of themes | Methods – “Deductive (SEIPS) and inductive coding” |
| 27 | Software | Methods – NVivo used for coding; Excel used for quotes |
| 28 | Participant checking | Methods – “PPI contributors reviewed preliminary findings; participants did not directly check themes” |
| 29 | Quotations presented | Results – illustrative quotations with participant IDs provided throughout |
| 30 | Data and findings consistent | Results – themes supported by multiple quotations; cross-checked by team |
| 31 | Clarity of major themes | Results – Five major themes clearly presented: release practices, lack of care coordination, staffing issues, transfer of information, patient factors |
| 32 | Clarity of minor themes | Results – sub-themes and system-level interventions described with examples |
