# Supplementary material for: Exploring Medication Safety in Transitions From Prison to Community: A Qualitative Study
Source: Health Expect. 2026 May 3;29(3):e70684. doi: 10.1111/hex.70684 (PMC13136601; doi:10.1111/hex.70684)
Supplement: Supplementary file 2 — Supporting File 2: [file HEX-29-e70684-s001.docx]

**Prisons Transitions Interview Schedule**

**Interview Overview**

- Thank you for agreeing to talk to me today. The purpose of the interview is to discuss the medication issues that can occur when people leave prison and return to the community. The overall aim of the study is to understand the nature and causes of these issues and identify ways to prevent issues from occurring in the future.
- If you agree, this interview will be recorded. The recording will be treated with the strictest confidence and may be listened to by members of the research team but nobody else. The recording will not be labelled with your name and anything that is written down based on it will be pseudonymised.
- If at any point I mention something you do not want to talk about, just let me know and we can move on to something else – you do not have to talk about anything that you do not want to. You can also stop the interview at any point without giving a reason.

**For face-to-face interviews**

- Have you had the opportunity to read the participant information sheet; do you have any questions about the information sheet or the study in general?
- Please read each section on the consent form and if you agree to the statement and are happy to provide consent for that aspect please initial the box. If you want me to read the statements out to you just let me know.
- You will keep one copy of the consent form and I will keep the other.
- Researcher provides the consent forms for signing
- Do you have any final questions before we begin?

**For telephone/ online interviews**

- Have you had the opportunity to read the participant information sheet; do you have any questions about the information sheet or the study in general?
- For each section on the consent form I will ask you to provide **verbal** consent. If you are happy to consent please say ‘yes I give my consent’.
- You will keep a copy of the unsigned-consent form along with the participant information sheet and I will securely store the recording of your consent.
- Researcher now reads through the consent form and takes verbal consent
- Do you have any final questions before we begin?

**Key safety issues**

- In your opinion, what are the key medication safety issues that occur when prisoners are released from prison back to the community?
- Thank you, I have made a note of these points and we will return to them later

**Exploring safety issues using SEIPS**

*We are now going to spend some time discussing the safety issues that you mentioned in the first part of the interview.*

- Did the safety issue [name issue] you are talking about involve you or another professional(s)?

If participant knows the details use prompts below.

- When did the [safety issue] occur i.e., time of day, month, transitions point
- What was the exact setting of the [safety issue] you are talking about? (*if not mentioned*)
- Please could you describe exactly what happened and why you believe it to be a safety issue (prompting start of ‘story’)

*Use prompts (using SEIPS) below to use while exploring what happened. Explore if any elements related to each other e.g. lack of communication due to physical environment. Mark under each category when needed as a reminder to prompt further questions.*

| **SEIPS Element** | | |
| --- | --- | --- |
| **Person**  Education, skills and knowledge; Motivation and needs i.e., complexities with preexisting condition. Physical/psychological characteristics | **Tasks**  Capacity and demand, level of task complexity, distractions; interruptions; variety of tasks; job content, challenge and utilization of skills; autonomy, job control and participation; job demands (e.g. workload, time pressure, cognitive load, need for attention) | **Tools & Technology**  Design interaction and usability issues; positioning; availability; access; mobility; operational/calibrated; device usability; various IT design issues; electronic records, barcoding. |
|  |  |  |
| **Physical Environment**  Layout; Noise; Lighting; temperature; humidity and air quality; design of immediate workspace or physical environment layout; location; size; clutter; standardisation, aesthetics; crowding. | **Organization of Work**  Teamwork. Length of clinic visits  Coordination, collaboration and communication  Work schedules, Social relationships  Supervisory and management style  Performance evaluation, rewards and incentives | **External Influences**  Societal, government, cultural, accreditation and regulatory influences e.g. funding, national policies and targets, professional bodies, regulatory demands, legislation and legal influences |
|  |  |  |

**Exploring safety issues CONT**

- Did this impact the patient’s physical or mental health or wellbeing?
   (i.e., short term impact; long term impact; any knock-on effect). Please could you explain how?
- Could this [safety issue] have been avoided? If so, could you describe how?
- Do you feel like this is possible? Could you explain why?
- Do you think this [safety issue] was an isolated incident or a reoccurring problem?

**Handling the issue**

- Were you able to speak to anyone about the [safety issue]?
  - **If not**, is there anything that is preventing you from doing so?

(*did you know this could be reported/is this the first time you are mentioning it?*)

- Has the safety concern been resolved? Or do you feel that steps are being put in place to satisfactorily resolve the issue?
  - **If not:** is there anything which you feel is preventing the issue from being resolved?
- Did you expect or receive any further support since this issue occurred? If yes, why did you expect that or how did you receive that? How helpful or unhelpful was the support? What more could have been done?

**Closing**

Is there anything else that we have not covered which you think might be useful for us to know?

*Thank you very much for taking part in this interview today, we really appreciate your time to talk to us.*

**Reflexivity questions (for interviewer’s post interview)**

- What went well during interview?
- Anything in particular that helped?
- What did not go so well?
- Are there any changes needed to the interview schedule?
- How did you feel the SEIPS prompts worked during the interview? Was there anything that did not work so well?
- Any questions missing/any other prompts needed?
