# Supplementary material for: Exploring Medication Safety in Transitions From Prison to Community: A Qualitative Study
Source: Health Expect. 2026 May 3;29(3):e70684. doi: 10.1111/hex.70684 (PMC13136601; doi:10.1111/hex.70684)
Supplement: Supplementary file 3 — Supporting File 3: [file HEX-29-e70684-s002.docx]

**Appendix 2: Participant role table**

| **Participant ID** | **Role** |
| --- | --- |
| P1 | Clinical pharmacist (prisons) |
| P2 | Nurse (prisons) |
| P3 | Clinical pharmacist (prisons) |
| P4 | Clinical pharmacist (prisons) |
| P5 | Community pharmacist |
| P6 | GP |
| P7 | Prisons officer |
| P8 | Community pharmacist |
| P9 | GP |
| P10 | Nurse (prisons) |
| P11 | Clinical pharmacist (prisons) |
| P12 | Prisons officer |
